# Intracorporeal versus extracorporeal urinary diversion in robot-assisted radical cystectomy: a systematic review and meta-analysis

**DOI:** 10.1007/s10147-021-01972-2

**Published:** 2021-06-19

**Authors:** Satoshi Katayama, Keiichiro Mori, Benjamin Pradere, Hadi Mostafaei, Victor M. Schuettfort, Fahad Quhal, Reza Sari Motlagh, Ekaterina Laukhtina, Marco Moschini, Nico C. Grossmann, Yasutomo Nasu, Shahrokh F. Shariat, Harun Fajkovic

**Affiliations:** 1 Department of Urology, Comprehensive Cancer Center, Vienna General Hospital, Medical University of Vienna, Währinger Gürtel 18-20, 1090 Vienna, Austria; 2 Department of Urology, Okayama University Graduate School of Medicine, Dentistry and Pharmaceutical Sciences, Okayama, Japan; 3 Department of Urology, The Jikei University School of Medicine, Tokyo, Japan; 4 Department of Urology, University Hospital of Tours, Tours, France; 5 Research Center for Evidence Based Medicine, Tabriz University of Medical Sciences, Tabriz, Iran; 6 Department of Urology, University Medical Center Hamburg-Eppendorf, Hamburg, Germany; 7 Department of Urology, King Fahad Specialist Hospital, Dammam, Saudi Arabia; 8 Men’s Health and Reproductive Health Research Center, Shahid Beheshti University of Medical Sciences, Tehran, Iran; 9 Institute for Urology and Reproductive Health, Sechenov University, Moscow, Russia; 10 Department of Urology, Luzerner Kantonsspital, Luzern, Switzerland; 11 Department of Urology, University Hospital Zurich, Zurich, Switzerland; 12 Department of Urology, Weill Cornell Medical College, New York, NY USA; 13 Department of Urology, University of Texas Southwestern, Dallas, TX USA; 14 Department of Urology, Second Faculty of Medicine, Charles University, Prague, Czech Republic; 15 Karl Landsteiner Institute of Urology and Andrology, Vienna, Austria; 16 Division of Urology, Department of Special Surgery, Jordan University Hospital, The University of Jordan, Amman, Jordan

**Keywords:** Robot-assisted radical cystectomy, Intracorporeal urinary diversion, Extracorporeal urinary diversion, Complication, Hospital volume, Meta-analysis

## Abstract

This systematic review and meta-analysis aimed to assess and compare the perioperative and oncological outcomes of intracorporeal (ICUD) and extracorporeal (ECUD) urinary diversion following robot-assisted radical cystectomy (RARC). A systematic literature search of articles was performed in PubMed^®^, Web of Science^®^, and Scopus^®^ databases according to the Preferred Reporting Items for Systematic Review and Meta-Analysis statement. We included studies that compared patients who underwent RARC with ICUD to those with ECUD. Twelve studies including 3067 patients met the eligibility criteria. There were no significant differences between ICUD and ECUD in overall and major complications, regardless of the period (short-term [≤ 30 days] or mid-term [> 30 days]). Subgroup analyses demonstrated that ICUD performed by high-volume centers exhibited a significantly reduced risk of major complications (short-term: OR 0.57, 95% CI 0.37–0.86, *p* = 0.008, mid-term: OR 0.66, 95% CI 0.46–0.94, *p* = 0.02). Patients who underwent ICUD had lower estimated blood loss (MD -102.3 ml, 95% CI − 132.8 to − 71.8, *p* < 0.00001), less likely to receive blood transfusion rates (OR 0.36, 95% CI 0.20–0.62, *p* = 0.00003); and these findings were consistent in subgroup analyses by low-volume centers (MD-121.6 ml, 95% CI − 160.9 to − 82.3, *p* < 0.00001 and OR 0.36, 95% CI 0.20–0.62, *p* = 0.00003, respectively). ICUD had a higher lymph node yield (MD 3.68, 95% CI 0.80–6.56, *p* = 0.01). Patients receiving ICUD provided comparable complications, superior perioperative outcomes, and similar oncological outcomes compared with ECUD. Centralization of patients may contribute to a reduction of postoperative complications, while maintaining the advantages.

**Supplementary Information:**

The online version contains supplementary material available at 10.1007/s10147-021-01972-2.

## Introduction

Radical cystectomy with pelvic lymph node (LN) dissection is the mainstay of treatment for patients with muscle-invasive bladder cancer and very high-risk non-muscle-invasive bladder cancer [1, 2]. Since its advent in 2003, the adoption of robot-assisted radical cystectomy (RARC) has increased steadily worldwide with the promise to lower complications and improve perioperative outcomes, while receiving oncologic efficacy [3–7]. Randomized controlled trials (RCTs) revealed that RARC with extracorporeal urinary diversion (ECUD), the initial type of urinary diversion (UD), did not improve complication rates compared to open radical cystectomy (ORC) [8, 9]. ICUD has been introduced with the aim to fulfill the promise of RARC to reduce complications, including bowel occlusion due to limited manipulation and air exposure, wound-related complications, and anastomotic stricture [10, 11]. To date, some observational studies suggest an advantage to ICUD over EUCD [12, 13], but no randomized trial has yet compared the differences between these two types of UD. Indeed, due to the heterogeneity in patient population and surgical factors, such a trial will be challenging because of the needed sample size and randomization. Recently, the International Robotic Cystectomy Consortium (IRCC) database, although this cohort consisted of 26 institutions, reported that intracorporeal urinary diversion (ICUD) use increased dramatically by an 11% increase rate per year, from 9% in 2005 to 97% in 2016 [14]. However, due to this data from leading experienced institutions, it may not represent real-world data. Since these retrospective studies are subject to inherent recruitment and allocation bias, it might not reflect the true impact of ICUD. One major concern for ICUD has caused by an increased risk of perioperative morbidity with prolonged operative time due to its highly complex procedure. Thereby, whether a wide variety of institutions should willingly incorporate ICUD at the cost of the great disadvantage remains unclear. For an understanding of the current situation in the impact of ICUD, we performed a systematic review and meta-analysis of the literature comparing complications as well as perioperative and oncological outcomes between ICUD and ECUD. Moreover, we evaluated the effect on hospital volume of this complex procedure by performing subgroup analyses. The provided data should help set up a framework for discussion and trial planning.

## Patients and methods

This study is registered with the International Prospective Register of Systematic Reviews (CRD: 42020212880).

### Literature search strategy

The present systematic review and meta-analysis were performed according to PRISMA (the Preferred Reporting Items for Systematic Reviews and Meta-Analyses) statement [15]. A comprehensive literature search using the electronic database (PubMed, Web of Science, and Scopus) was carried out in September 2020 to retrieve published articles comparing complications, perioperative and oncological outcomes of patients who underwent RARC with ICUD to those who underwent RARC with ECUD. The search for eligibility was independently performed by two authors using the following string terms: (urothelial carcinoma OR urothelial cancer OR bladder cancer OR bladder carcinoma) AND (robot-assisted radical cystectomy OR da Vinci radical cystectomy OR robot radical cystectomy) AND (diversion OR ileal conduit OR neobladder) AND (perioperative OR complication OR morbidity OR mortality). The primary outcomes were complications between ICUD and ECUD, graded with the Clavien–Dindo system into overall (grades 1–5) and major (grades 3–5). Concerning the observed period, we divided the complications into short-term (≤ 30 days) and mid-term (> 30 days). The secondary outcomes were perioperative outcomes, including operative time, estimated blood loss (EBL), blood transfusion rates, length of stay (LOS), ileus, gastrointestinal (GI)-related complications, and oncological outcomes, including LN yield, number of positive LNs, and soft tissue surgical margin (STSM). After a first screening based on the study title and abstract, the second screening was based on the full-text review and excluded with reasons when deemed inappropriate. Disagreements were resolved via a consensus with coauthors.

### Inclusion/exclusion criteria

The clinical question was established, as stated in the PICO (Population, Intervention, Comparator, Outcome, Study design) approach. Studies were included when bladder cancer patients (P) who had undergone RARC with ICUD (I) as compared with those who had undergone RARC with ECUD (C) in terms of complications, perioperative and oncological outcomes (O) using randomised controlled or observational cohort studies (S). In case of multiple publications on the same cohort, either the high quality or the most recent publication was selected. Reviews, letters, editorials, comments, meeting abstracts, case reports, and articles not published in English were excluded.

### Data extraction

Two authors independently conducted data extraction from the included articles. The extracted data included: first author’s name, publication year, period of patient recruitment, recruitment region, study design, number of patients, age, gender, body mass index, neoadjuvant chemotherapy, The American Society of Anesthesiology score, number of surgeons, operative time, EBL, blood transfusion rates, LOS, type of UD, overall and major complications, ileus, GI-related complications, pathologic stage, STSM, LN yield and positive LN. All discrepancies in the data extraction were resolved by consensus with co-investigators.

### Statistical analysis

Forest plots were used as the summary variables for dichotomous and continuous outcomes and to describe the relationships between different UDs and each outcome. Continuous variables are presented as mean ± standard deviation (SD), and compared with mean differences (MDs). Dichotomous variables are presented as proportions and compared with odds ratios (ORs) and 95% confidence intervals (CIs). Continuous variables reported as median and interquartile range were altered to mean and SD using the formulas given by Wan et al. [16]. We summarized data using a random-effect model as the studies were heterogeneous. Subsequently, we also performed subgroup analyses according to hospital volume in ICUD. A high-volume center was defined as studies included the number of patients receiving ICUD per hospital per year was ≥ 10, and a low-volume center was defined as the number of patients was < 10, as recommended by the European Association of Urology Muscle-invasive and Metastatic Bladder Cancer Guideline Panel (EAU MIBC panel) [17]. In the study from IRCC database [18], we estimated the number of patients who underwent ICUD before propensity score matching while referring to the previous study with the same database [14]. Subgroup analyses of complication rates were conducted in studies from high-volume centers as examined the impact of ICUD with reduced the effect of the learning curve. Subgroup analyses of perioperative outcomes were conducted in studies from low-volume centers as examined the adverse effects of ICUD with the learning curve strongly considered. Heterogeneity among the included studies' outcomes in the meta-analysis was evaluated using the *I*^2^ statistics and the Cochrane *Q* test. Significant heterogeneity was indicated by an *I*^2^ test greater than 50% and *p* < 0.05 in the Cochrane *Q* test. Publication bias was assessed using funnel plots. Statistical analyses were carried out using Review Manager 5.4 (The Nordic Cochrane Centre, Copenhagen, Denmark); the statistical significance level was *p* < 0.05.

### Risk of bias

Two authors independently evaluated the risk of bias for all prospective or retrospective cohort studies using the ROBINS-I tool [19]. All discrepancies regarding the risk of bias were resolved by consensus with coauthors.

## Results

### Search results

**Fig. 1:**
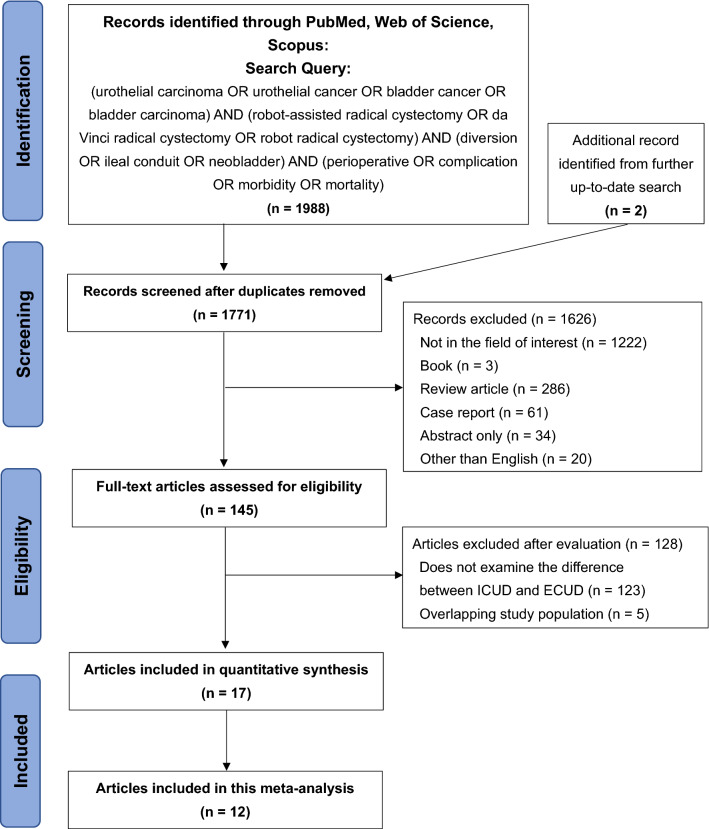
Flow diagram of the study selection procedure for the systematic review and meta-analysis. *ICUD* intracorporeal urinary diversion; *ECUD* extracorporeal urinary diversion

We identified 298 studies in PubMed, 1659 studies in Web of Science, and 31 studies in Scopus from the initial search; 2 additional studies were added after the latest search. After removing 219 duplicate articles, we screened 1771 titles and abstracts, reviewed 145 full-text articles, which led to the identification of 12 articles comprising 3067 patients that met our inclusion/exclusion criteria [18, 20–30]. Five articles were excluded because of an overlap in the recruitment period at the same institution (Fig. 1). Five and seven studies were divided, respectively, into high-volume center subgroup and low-volume center subgroup. The risk of bias is depicted in Supplementary Table 2.

### Included studies

Overall, 1401 patients (45.7%) underwent ICUD and 1666 (54.3%) underwent ECUD. Table 1 demonstrates the basic characteristics of the included studies. Complications, perioperative and oncological outcomes are summarized in Table 2 and Supplementary Table 1. All studies were non-randomized controlled studies. In seven studies (58.3%), RARC plus ICUD were performed by multiple surgeons. Mistretta et al. [30] compared orthotopic ileal neobladder performed intracorporeally vs extracorporeally. Zhang et al. [25] utilized enhance recovery after surgery (ERAS). Hussein et al. [18] used propensity score matching using data from IRCC.

**Table 1:** Baseline characteristics of the included studies

	Recruitment year	Region	Study design	No. patients ICUD/ECUD	Age, mean (y) ICUD/ECUD	Male (%)	BMI ICUD/ECUD	NAC rate (%) ICUD/ECUD	ASA score > 3 (%)	No. of surgeons	Hospital volume in ICUD, *n* (/y)
Pruthi et al. [28]	2008–2009	USA	Retrospective	12/20	61/67	75/70	28/27	NR	NR	NR	3.3
Aboumohamed et al. [29]	2009–2012	USA	Retrospective	42/120	70/68	79/72	31/28	NR	50/58.6	NR	10.5
Pyun et al. [26]	2007–2014	South Korea	Retrospective	26/38	65/63	92/92	25/25	23.1/18.4	NR	1	3.3
Kingo et al. [27]	2012–2015	Denmark	Prospective	38/12	68/68	82/83	27/24	34.2/0	5.3/16.7	5	9.5
Lenfant et al. [20]	2010–2016	France	Retrospective	74/34	67/68	81/94	26/26	41.9/50	14.9/41/2	5	2.1
Tan et al. [22]	2015–2017	UK	Retrospective	59/68	68/71	83/88	27/27	22/13.2	NR	NR	19.6
Bertolo et al. [21]	2014–2017	USA	Prospective	60/66	69/73	77/86	30/28	36.7/31.8	98.3/97.0	2	15
Hussein et al. [18]	2005–2018	USA	Retrospective	486/486	69/68	77/78	28/27	18.7/19.8	49.0/49.4	multiple	3.8
Mistretta et al. [30]	2014–2019	Italy	Retrospective	57/44	60/62	NR	27/27	49.1/20.5	NR	NR	9.5
Mazzone et al. [23]	2004–2018	Italy	Retrospective	162/105	71/68	83/85	26/26	24.1/25.7	32.1/40	3	10.8
Shim et al. [24]	2007–2017	South Korea	Retrospective	84/278	64/65	86/86	24/24	10.7/9.7	NR	6	7.6
Zhang et al. [25]	2011–2018	USA	Retrospective	301/375	68/69	77/83	27/28	36.2/37.1	NR	> 10	37.6

*ICUD* intracorporeal urinary diversion; *ECUD* extracorporeal urinary diversion, *BMI* body mass index, *NAC* neoadjuvant chemotherapy, *ASA* American Society of Anaesthesiology

**Table 2:** Perioperative complications of the included studies

	Short-term complication (overall) (%)	Short-term complication (major) (%)	Mid-term complication (overall) (%)	Mid-term complication (major) (%)	Ileus (%)	Gastrointestinal-related complication (%)	Wound-related complication (%)
Pruthi et al. [28]	42/40	NR	0/10	NR	NR	NR	NR
Aboumohamed et al. [29]	NR	NR	NR	NR	NR	NR	NR
Pyun et al. [26]	NR	NR	NR	NR	NR	0/5	0/11
Kingo et al. [27]	100/100	26/0	100/100	32/8	13/8	NR	0/0
Lenfant et al. [20]	47/38	9/6	19/29	12/18	NR	NR	NR
Tan et al. [22]	51/74	8/10	12/15	8/9	NR	NR	NR
Bertolo et al. [21]	22/14	2/2	7/6	2/0	5/9	10/17	0/2
Hussein et al. [18]	47/22	12/7	6/6	2/1	NR	23/16	13/9
Mistretta et al. [30]	58/59	19/20	42/36	28/25	9/7	NR	NR
Mazzone et al. [23]	35/43	NR	NR	NR	NR	NR	NR
Shim et al. [24]	NR	NR	NR	NR	NR	5/13	4/3
Zhang et al. [25]	38/43	10/18	44/48	17/25	21/27	23/29	NR

### Complications

#### Short-term complications

**Fig. 2:**
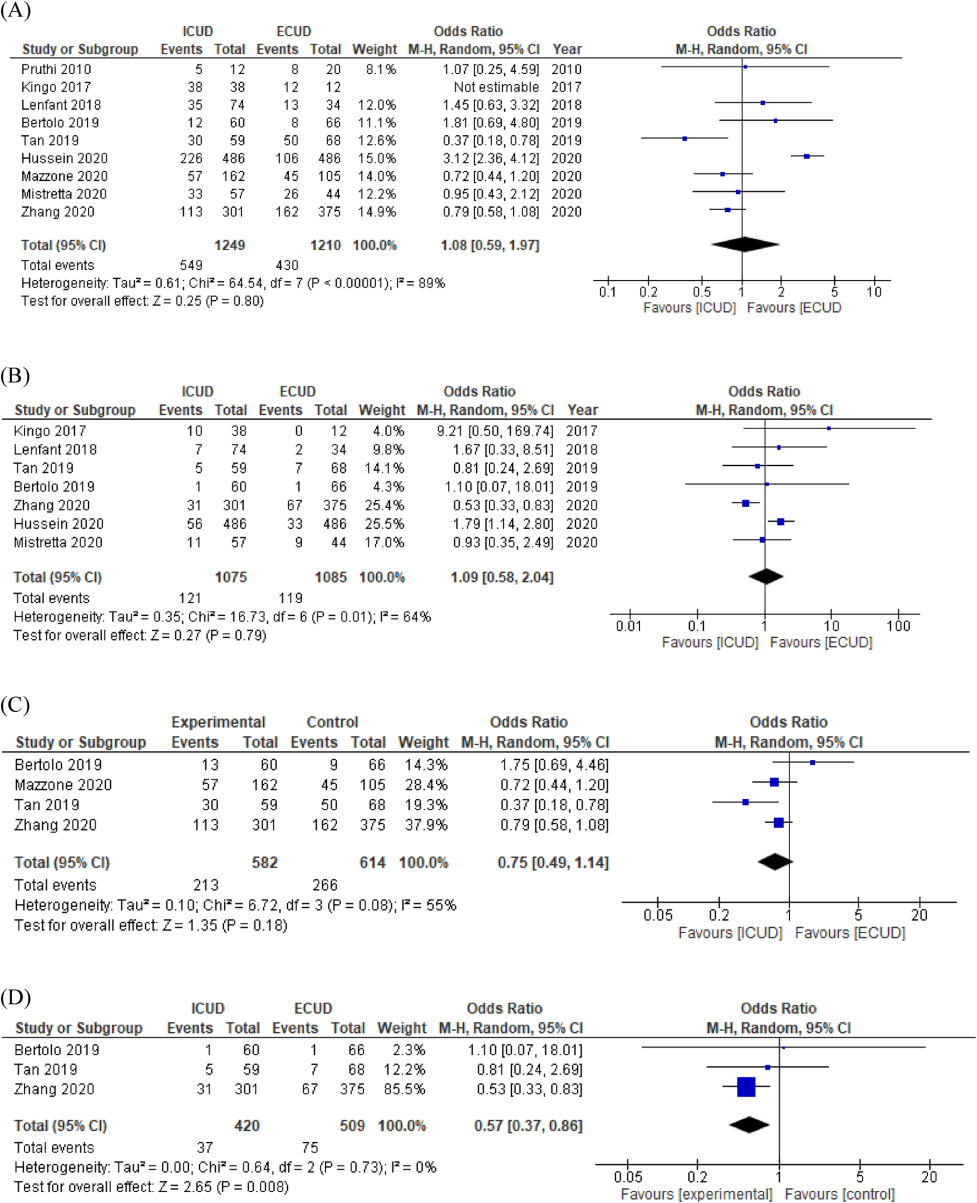
Forest plots of studies investigating the comparison of **A** short-term overall complication, **B** short-term major complication, and **C** subgroup of short-term overall complication, **D** subgroup of short-term major complication classified by hospital volume between RARC with ICUD and RARC with ECUD

Nine studies comprising 2459 patients and seven studies comprising 2160 patients were analyzed for overall and major complications, respectively. Forest plots (Fig. 2A, B) showed that there were no significant differences in overall and major complications between ICUD and ECUD (OR 1.08, 95% CI 0.59–1.97, *p* = 0.80 and OR 1.09, 95% CI 0.58–2.04, *p* = 0.79, respectively). Between studies, heterogeneities in overall and major complications were significant based on the Cochrane *Q* test (*p* < 0.00001 and *p* = 0.01, respectively) and *I*^2^ test (89% and 64%, respectively). A subgroup analysis of high-volume centers showed that there was no significant difference in overall (OR 0.75, 95% CI 0.49–1.14, *p* = 0.18) complications, but ICUD in high-volume centers was significantly associated with a reduced risk of major (OR 0.57, 95% CI 0.37–0.86, *p* = 0.008) complications (Fig. 2C, D). No significant heterogeneities were observed in these subgroup analyses (Cochrane *Q* test [*p* = 0.08 and *p* = 0.73] and *I*^2^ [55% and 0%] in overall and major complications, respectively). The funnel plots for overall and major short-term complications identified four and two studies over the pseudo-95% CI, respectively (Supplementary Fig. 3A, 3B).

#### Mid-term complications

**Fig. 3:**
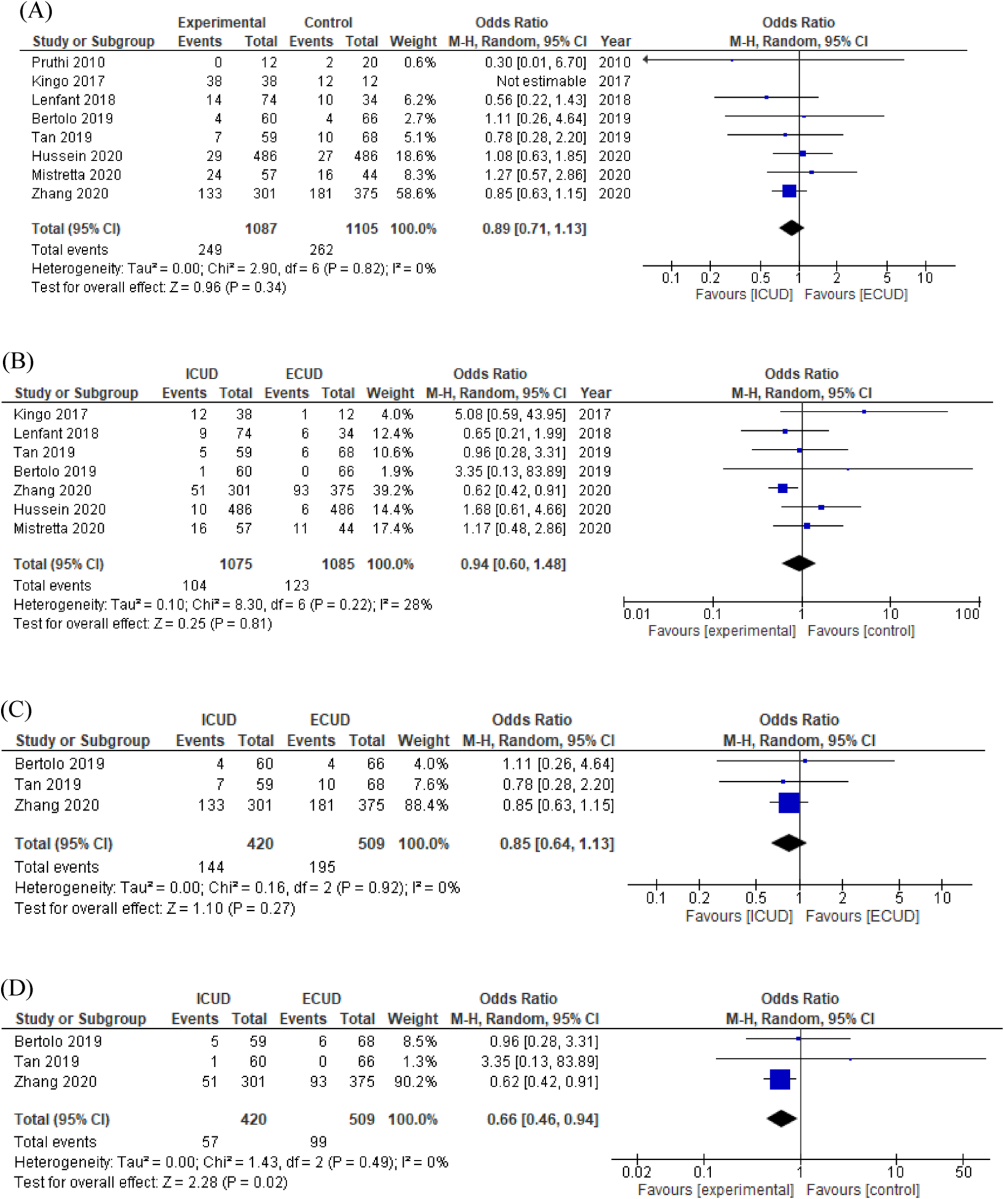
Forest plots of studies investigating the comparison of **A** mid-term overall complication, **B** mid-term major complication, and **C** subgroup of mid-term overall complication, **D** subgroup of mid-term major complication classified by hospital volume between RARC with ICUD and RARC with ECUD

Eight studies comprising 2193 patients and 7 studies comprising 2160 patients were analyzed for overall and major complications, respectively. The forest plots (Fig. 3A, B) showed that there were no significant differences in overall and major complications (OR 0.89, 95% CI 0.71–1.13, *p* = 0.34 and OR 0.94, 95% CI 0.60–1.48, *p* = 0.81, respectively). Cochrane *Q* test (*p* = 0.82 and *p* = 0.22, respectively) and *I*^2^ test (0% and 28%) showed no significant heterogeneities. A subgroup analysis of high-volume centers showed that there was no significant difference in overall (OR 0.85, 95% CI 0.64–1.13, *p* = 0.27) complications, but ICUD in high-volume centers was significantly associated with a reduced risk of major (OR 0.66, 95% CI 0.46–0.94, *p* = 0.02) complications (Fig. 3C, D). The funnel plot in both overall and major complications identified no study over the pseudo-95% CI (Supplementary Fig. 3C, 3D).

### Perioperative outcomes

**Fig. 4:**
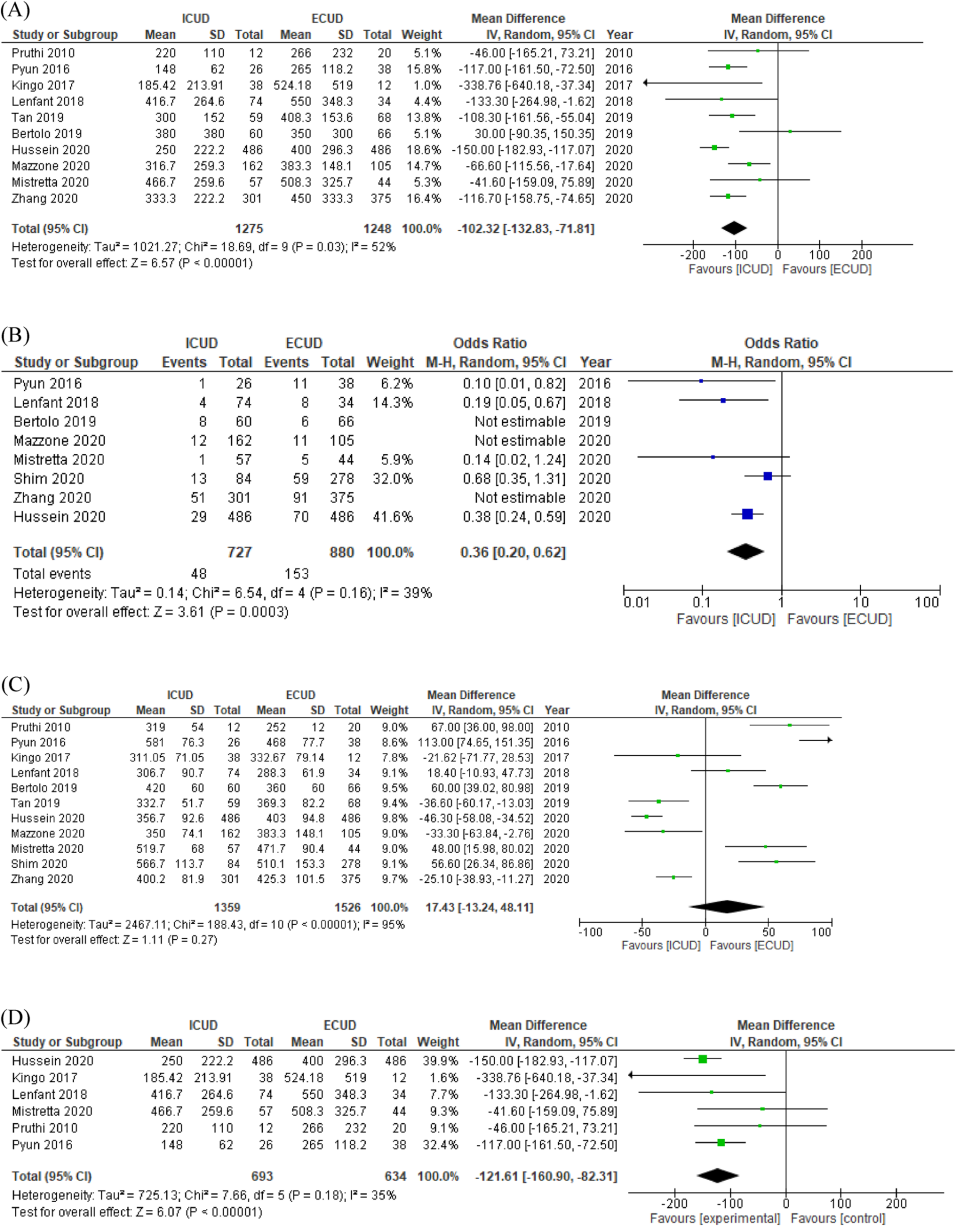

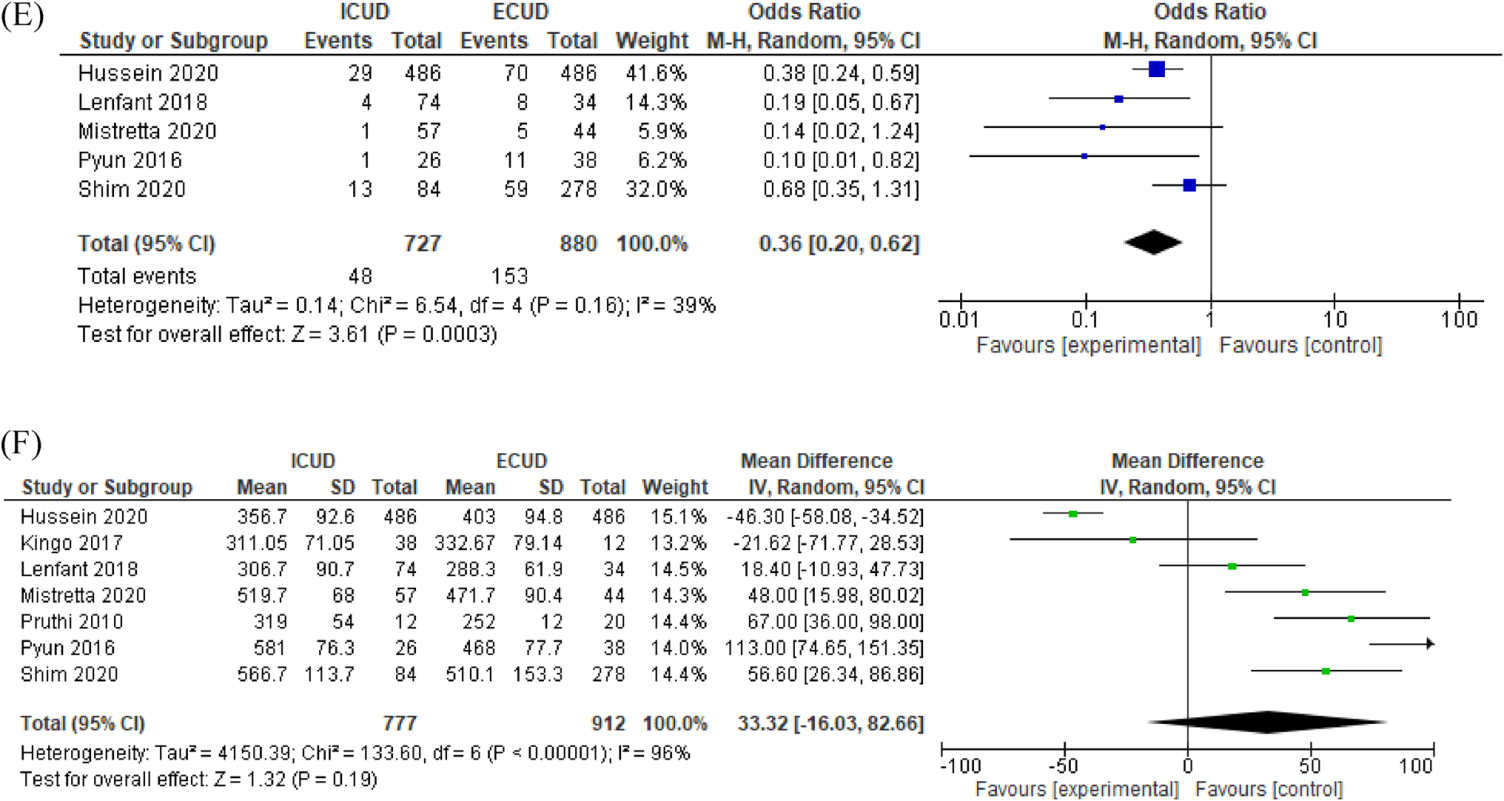
Forest plots of studies investigating the comparison of **A** estimate blood loss, **B** blood transfusion rates, **C** operative time, and **D** subgroup of estimate blood loss, **E** subgroup of blood transfusion rates, **F** subgroup of operative time classified by hospital volume between RARC with ICUD and RARC with ECUD

EBL and blood transfusion rates were significantly lower in patients who underwent an ICUD (MD −102.3 ml, 95% CI −132.8 to −71.8, *p* < 0.00001 and OR 0.36, 95% CI 0.20–0.62, *p* = 0.00003, respectively) compared to those who underwent an ECUD (Fig. 4A, B). Operative time and LOS were not significantly different between ICUD and ECUD (MD 17.4 min, 95% CI −13.2 to 48.1, *p* = 0.27 and MD −0.87, 95% CI −2.12 to 0.39, *p* = 0.17, respectively) (Fig. 4C, Supplementary Fig. 1A). Ileus exhibited a marginally trend toward benefit for ICUD but this did not reach statistical significance (OR 0.72, 95% CI 0.53–1.03, *p* = 0.07) (Supplementary Fig. 1B). There was no difference in GI-related complications between the two UDs (OR 0.75, 95% CI 0.41–1.39, *p* = 0.36) (Supplementary Fig. 1C). A subgroup analysis of low-volume centers showed that EBL and blood transfusion rates remained significantly different (MD −121.6 ml, 95% CI −160.9 to −82.3, *p* < 0.00001 and OR 0.36, 95% CI 0.20–0.62, *p* = 0.00003, respectively) (Fig. 4D, 4E). Subsequently, operative time and LOS also remained no significantly different in subgroup analyses of low-volume centers (MD 33.3 min, 95% CI −16.0 to 82.7, *p* = 0.19 and MD -0.21, 95% CI −2.46 to 2.04, *p* = 0.86, respectively) (Fig. 4F, Supplementary 2D). The funnel plots identified two studies for EBL, no study for blood transfusion rates, seven studies for operative time, and five studies for LOS over the pseudo-95% CI (Supplementary Fig. 3E–H, respectively).

### Oncological outcomes

Patients receiving an ICUD had a significantly higher LN yield than those who received an ECUD (MD 3.68, 95% CI 0.80–6.56, *p* = 0.01) (Supplementary Fig. 2A). STSM and the positive LN were not significantly different between ICUD and ECUD (OR 1.02, 95% CI 0.74–1.40, *p* = 0.92 and OR 1.12, 95% CI 0.79–1.59, *p* = 0.54, respectively) (Supplementary Fig. 2B, 2C). The funnel plots identified four studies for LN yield, no study for STSM, and one study for positive LN over the pseudo-95% CI (Supplementary Fig. 3I, 3J, 3K, respectively).

## Discussion

We performed a systematic review and meta-analysis to evaluate the clinical safety and efficacy of RARC with ICUD compared to ECUD by assessing complications, perioperative and oncological outcomes. Perioperative complications, including overall or major, short-term or mid-term, were comparable between ICUD and ECUD. Subgroup analyses suggested that patients receiving ICUD in high-volume centers had significantly a reduced risk of major complications, but not that of overall complications. Moreover, we found that patients who underwent an ICUD had a significantly lower EBL, lower transfusion rates; these findings remained even in patients from low-volume centers.

RC is a technically complex procedure that comprises two major steps: the bladder extirpation phase and the urinary reconstructive phase. With the growing popularity of RARC, the quest for improvement of outcomes has shown some evidence toward better outcomes with ICUD compared to ECUD while maintaining long-term survival outcomes and similar recurrence patterns as well as superior perioperative outcomes and comparable oncological outcomes compared to ORC [31–34]. It is well-known that the urinary reconstructive phase is the major driver of morbidity. By performing the UD intracorporeally, it has been argued that the expected benefits of complete pneumoperitoneum and minimal invasive surgery would lead to tangible improvement for RC patients. Indeed, others and we hypothesized that ICUD might have a positive impact in terms of postoperative complications compared to ECUD. We found, however, no difference in overall and major complications between ICUD and ECUD in our meta-analysis. For this reason, it may be conceivable that heterogeneity in the learning curve proficiency in the reconstructive phase affected these analyses. To flatten the learning curve in the extirpation phase, at least 30 cases are needed, which leads to reduced EBL, less STSM, or an adequate number of retrieved LN [35, 36]. However, it may be argued that the reconstructive phase needs a much longer learning curve to obtain the best possible complication rates compared to the extirpative phase [36]. Most urologists initially adopt ECUD, as they were familiar with this technique from the open approach. Over the years, the lack of significant benefit offered by ECUD resulted in a push to transition to ICUD. In this regard, almost all studies included in this meta-analysis represented the results comparing the first ICUD cases to the last ECUD cases. Considering that RARC is not a high-volume surgery, unlike robot-assisted radical prostatectomy, it appears to be evident that hospital volume was associated with perioperative complications, mortality, and long-term oncologic outcomes [17, 37]. Thus, EAU MIBC panel recently advocated a threshold number of RCs per hospital and/or surgeon (at least 10, and preferably > 20), being highlighted the potential benefit of centralization of RARC [17]. Despite a lack of high level evidence, further centralization of ICUD may be needed due to its technically complex aspect. Our subgroup analyses showed that RARC plus ICUD in high-volume centers was likely associated with a decreased incidence of postoperative major complications. This finding might exhibit the true impact of ICUD after the learning curve because the matured robotic team, including a skilled mentor and expertise in the patient care, help to shorten or mitigate the effect of the learning curve.

We found that EBL and blood transfusion rates were significantly lower in patients who received an ICUD rather than those who underwent an ECUD in the present study. Recent meta-analyses comparing RARC with ECUD vs. ORC described significantly lower EBL and blood transfusion rates in RARC than in ORC [5, 7, 33, 34], with this tendency being extended in RARC with ICUD in this study. This could be explained by the influence of pneumoperitoneum throughout the surgery and precise dissection due to three-dimensional magnified visualization. Interestingly, even in subgroup analyses of low-volume centers, statistically significant differences regarding EBL and blood transfusion retained between ICUD and ECUD, suggesting that the influence of pneumoperitoneum may outweigh a negative effect on the early phase of the learning curve. Blood transfusion has been shown to be significantly associated with worse perioperative morbidity and survival [38–40]. It was also noteworthy that ICUD patients with high age-adjusted Charlson Comorbidity Index had a lower risk of complications relative to ECUD patients [23]. One possible explanation for this could be that ICUD reduced surgical stress, including less blood loss, few transfusion rates, or less incision pain. As such, ICUD may possibly be even more indicated for older or frail patients [41].

Another potential benefit of ICUD is the faster recovery of bowel function due to the avoidance of excessive bowel manipulation and less exposure time to external air. The current meta-analysis showed that ileus was trending in favor of ICUD but failed to prove the statistical significance. The effect of ERAS on outcomes may have corrected for the background noise to assess differences between the UD types. ERAS pathways aim to minimize the physiological stress and facilitate postoperative recovery, which has led to improved perioperative outcomes, including shorter LOS and fewer GI-related complications [42, 43]. A small incision in ICUD may enable less use of opioids, and it is possible that the combination of ICUD and ERAS may provide further improvements in the rate of ileus through synergistic effects [44].

Although ICUD has often been criticized as a time-consuming procedure, we found no difference between ICUD and ECUD in operative time. Note that even when there was no significant difference in operative time by a subgroup analysis of low-volume centers, significant heterogeneity remains to be observed. Thus, in view of unknown cause of heterogeneity possibly occurred from inter-study, interpretation should be cautious. In addition, LN yields were significantly higher in patients treated with ICUD. This suggests a higher surgical proficiency of surgeons preferring ICUD in the published data to date. The determination of the operating technique (intracorporeal or extracorporeal) and UD (ileal conduit or neobladder) is multifactorial. It depends on the patient’s and surgeon’s preferences as well as the tumor pathologic aspect [45]. Experienced surgeons are likely to choose the most challenging but best possible surgery (i.e., ICUD) which may deliver a more favorable operative time and perioperative outcomes, including blood transfusion rates, EBL, and LN yields.

This systematic review and meta-analysis has some limitations. First, no RCTs were included in this study, which caused considerable selection bias. As mentioned above, the patient’s and surgeon’s preferences highly affect UD's decision-making. Second, there is some degree of heterogeneity in measuring outcomes. To standardize the reporting methodology, most of the studies reported complications using the Clavien–Dindo system, but few studies reported by the formed reporting system, which was recommended by an ad hoc European Association of Urology panel [46]. Third, despite subgroup analyses stratified by hospital volume, it may not reflect reality. Although IRCC undoubtedly represents a pioneer in the field of robotic surgeries, it consists of 26 centers combining academic and private centers, having been assigned to the low-volume center [25]. Additionally, this study highly affects our several analyses due to a large number of included patients but did not adjust any potential confounding factors inherent in a retrospective design, leading to confound the analyses. Fourth, although the extent of LN dissection is more clinically relevant than LN yields, we could not discuss about the extent of LN dissection due to lack of data in eligible studies. In addition, the cumulative experience of ICUD for each surgeon before the analysis is not unknown. Case selection is another bias that remains uncontrolled for. Finally, since RC is a complex procedure, late complications such as stenosis of uretero-ileal anastomosis are often concerns that remain unassessed. Unfortunately, there are very few available studies on this matter [10].

Beyond the limitations, our meta-analysis has practical implications. Since ICUD is a highly complex procedure, there appears to exist a long learning curve. However, at the cost of the learning curve and prolonged operative time, the data suggest that some perioperative benefits can be obtained for the patients. Shared knowledge, mentorship programs with surgeons and the development of a dedicated team organized around anesthesiologists, intensive care unit staffs and bedside assistants are needed to obtain on the potential benefits of ICUD.

In conclusion, this meta-analysis suggested that complications of RARC with ICUD in the short-term and mid-term periods were equivalent to those of ECUD with a trend toward faster bowel recovery. ICUD performed by high-volume centers significantly achieved the reduced risk of postoperative major complications. Furthermore, blood loss and transfusion rates were superior in patients receiving ICUD compared to those receiving ECUD, regardless of hospital volume. Based on these findings, centralization of patients who are candidates for ICUD seems advisable. The best treatment strategies for each individual patient at each institution should be determined, considering patient comorbidities, surgeon’s experience and hospital volume. However, since the choice of UD is likely to be subject to a strong bias, interpretation should be cautious. A trial comparing RARC with ICUD and ORC (iROC; clinicaltrials.gov: NCT03049410) in progress is awaited.

## Supplementary Information

Below is the link to the electronic supplementary material.

Supplementary file1 (DOCX 21 KB)

Supplementary file2 (DOCX 183 KB)
