# Supplementary material for: Intracorporeal versus extracorporeal urinary diversion in robot-assisted radical cystectomy: a systematic review and meta-analysis
Source: Int J Clin Oncol. 2021 Jun 19;26(9):1587–99. doi: 10.1007/s10147-021-01972-2 (PMC8364906; doi:10.1007/s10147-021-01972-2)
Supplement: Supplementary file 1 — Supplementary file1 (DOCX 21 KB) [file 10147_2021_1972_MOESM1_ESM.docx]

**Supplementary Table 1.**Perioperative and oncologic outcomes of the included studies

|  | Operative time  (mean) | EBL, mean (ml) | Blood transfusion  rates (%) | Length of stay (mean) | Ileal conduit (%) | ≥pT3 (%) | STSM (%) | LN yield | No. of Positive LN |
| --- | --- | --- | --- | --- | --- | --- | --- | --- | --- |
| Pruthi, 2010 | 319/252 | 220/266 | NR | 4.5/5.2 | 75/65 | NR | NR | NR | NR |
| Aboumohamed, 2014 | 363/366 | 410/490 | NR | 11/15 | 100/100 | NR | NR | NR | 4/22 |
| Pyun, 2016 | 581/468 | 148/265 | 4/29 | 15/16.7 | 58/61 | 19/21 | 0/0 | 32/23 | NR |
| Kingo, 2017 | 311/333 | 185/524 | NR | 10.9/8.2 | 100/100 | 13/25 | NR | 20/17 | NR |
| Lenfant, 2018 | 307/288 | 417/550 | 5/24 | 13.3/12 | 47/82 | NR | 8/6 | 15/14 | 14/0 |
| Tan, 2019 | 333/369 | 300/408 | NR | 8.3/8.3 | 100/100 | 32/37 | 8/7 | 16/16 | 10/11 |
| Bertolo, 2019 | 420/360 | 380/350 | 13/9 | 7/8 | 100/100 | 38/39 | 5/5 | 12/11 | 12/9 |
| Hussein, 2020 | 357/403 | 250/400 | 6/14 | 9.7/8.3 | 91/74 | 40/39 | 8/9 | 20/21 | 107/117 |
| Mistretta, 2020 | 520/472 | 467/508 | 2/11 | 12.7/12.3 | 0/0 | 28/30 | 0/0 | 25/18 | 12/6 |
| Mazzone, 2020 | 350/383 | 317/383 | 7/10 | 12.2/13.3 | 90/91 | 27/40 | 7/8 | 16/14 | 19/23 |
| Shim, 2020 | 567/510 | NR | 15/21 | 16.6/22.4 | 37/40 | 33/31 | 2/3 | 30/15 | 22/51 |
| Zhang, 2020 | 400/425 | 333/450 | 17/24 | 6/7.7 | 85/71 | 33/28 | 6/4 | NR | 64/56 |

EBL: estimate blood loss, STSM: soft tissue surgical margin, LN: lymph node

**Supplementary Table 2.** Risk of bias assessment for individual studies using the Risk of Bias in non-randomized controlled studies of interventions tool (ROBINS-I)

| **Author** | **Confounding** | **Participant selection** | **Classification of interventions** | **Deviation from intended intervention** | **Missing data** | **Measurement of outcomes** | **Selection of the**  **reported result** | **Overall** |
| --- | --- | --- | --- | --- | --- | --- | --- | --- |
| Pruthi 2010 | Serious | Serious | Low | Moderate | Low | Serious | Low | Serious |
| Aboumohamed 2014 | Serious | Moderate | Low | Moderate | Moderate | Low | Low | Serious |
| Pyun 2016 | Serious | Serious | Low | Moderate | Moderate | Serious | Low | Serious |
| Kingo 2017 | Serious | Serious | Low | Serious | Serious | Moderate | Low | Serious |
| Lenfant 2018 | Serious | Serious | Low | Serious | Low | Moderate | Low | Serious |
| Tan 2019 | Serious | Serious | Low | Moderate | Low | Moderate | Low | Serious |
| Bertolo 2019 | Moderate | Moderate | Low | Moderate | Low | Low | Low | Moderate |
| Hussein 2020 | Low | Low | Low | Low | Moderate | Moderate | Low | Moderate |
| Mistretta 2020 | Serious | Moderate | Low | Serious | Low | Moderate | Low | Serious |
| Mazzone 2020 | Moderate | Moderate | Low | Moderate | Moderate | Low | Low | Moderate |
| Shim 2020 | Serious | Low | Low | Moderate | Low | Moderate | Moderate | Serious |
| Zhang 2020 | Serious | Moderate | Low | Serious | Low | Moderate | Low | Serious |
