# Supplementary figures and images for: Intracorporeal versus extracorporeal urinary diversion in robot-assisted radical cystectomy: a systematic review and meta-analysis
Source: Int J Clin Oncol. 2021 Jun 19;26(9):1587–99. doi: 10.1007/s10147-021-01972-2 (PMC8364906; doi:10.1007/s10147-021-01972-2)

**Supplementary Fig. 1**


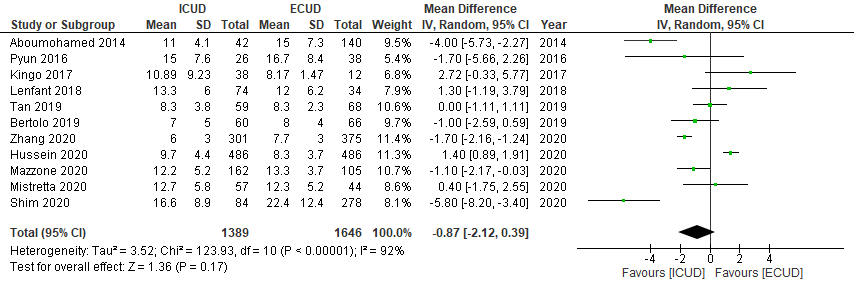


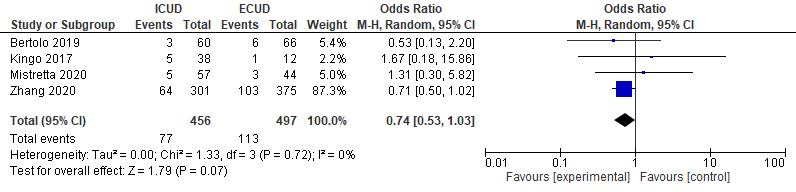


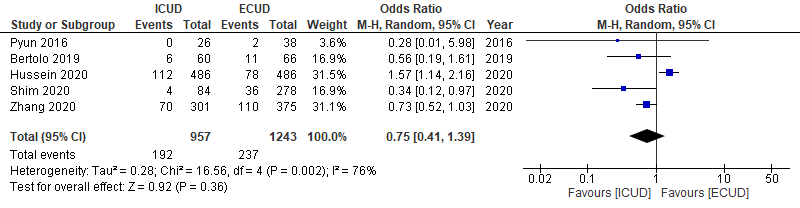


**
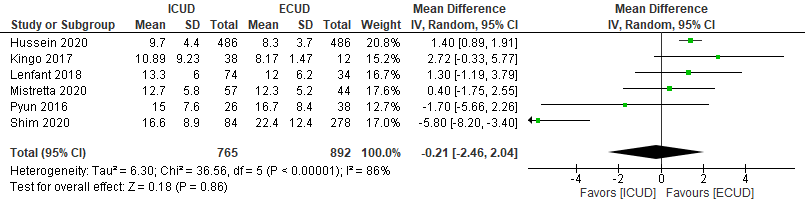
**

**Supplementary Fig. 2**


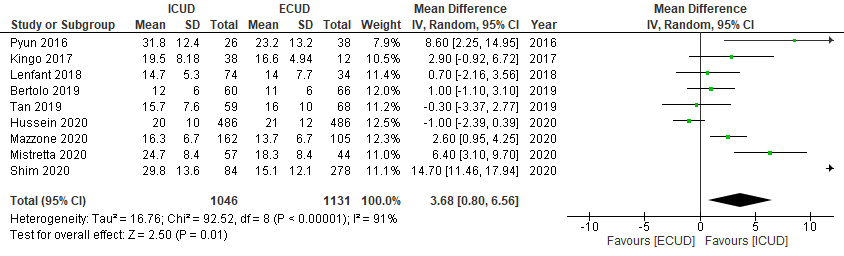


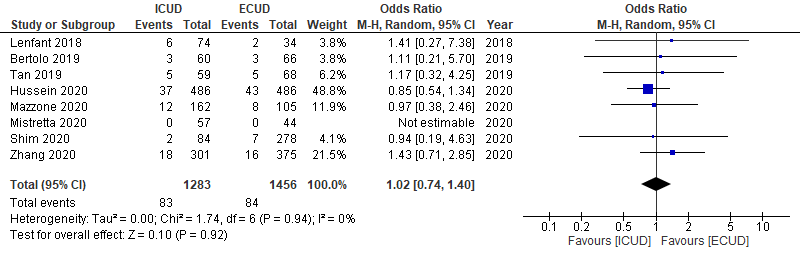


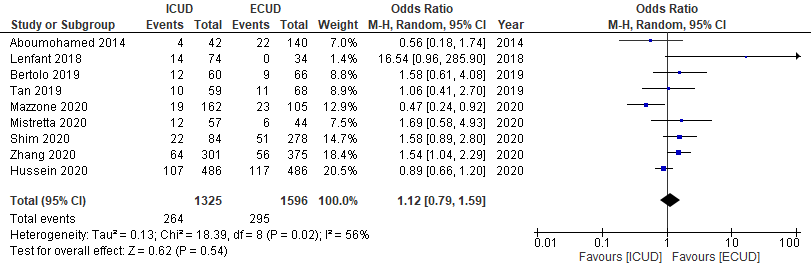


**Supplementary Fig. 3**


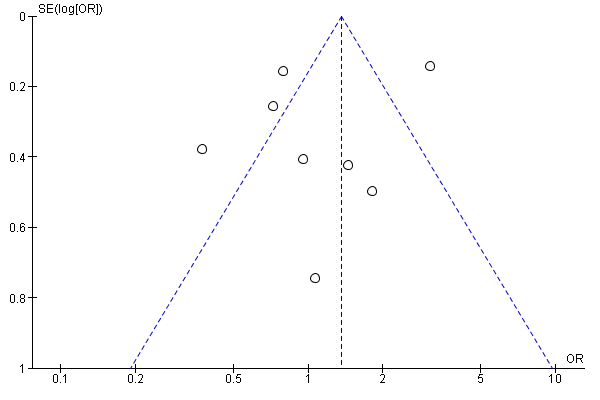


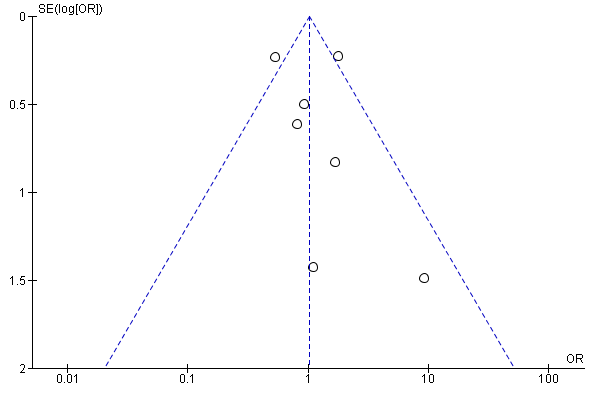


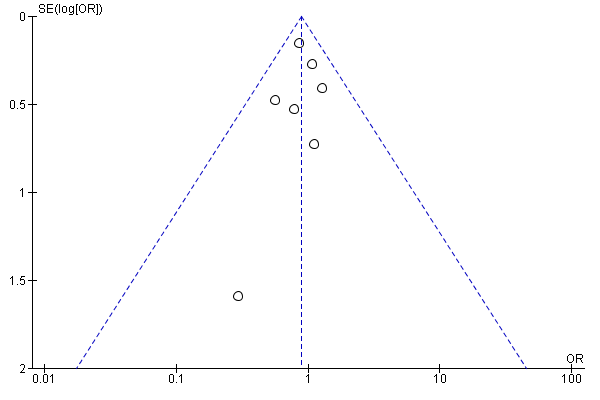


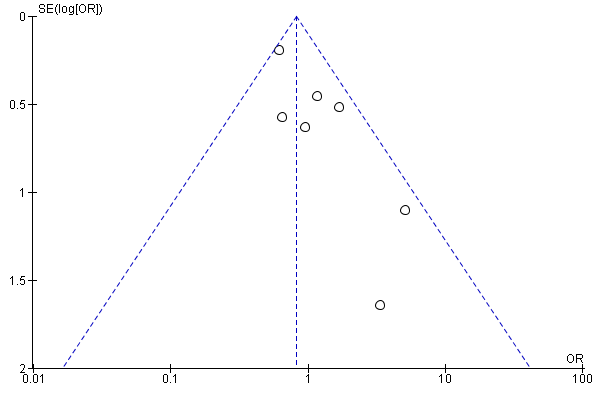


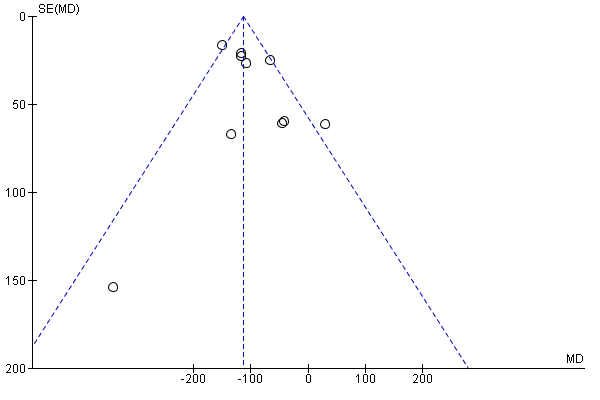


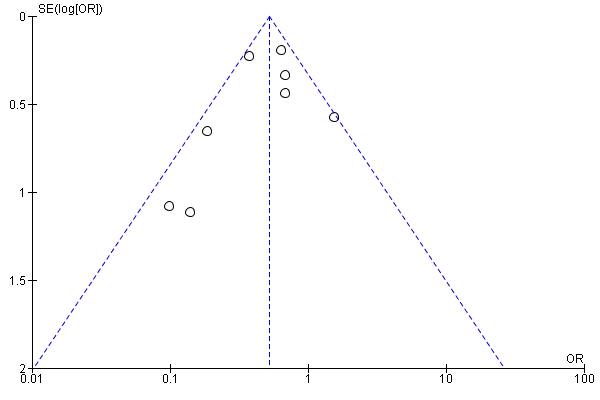


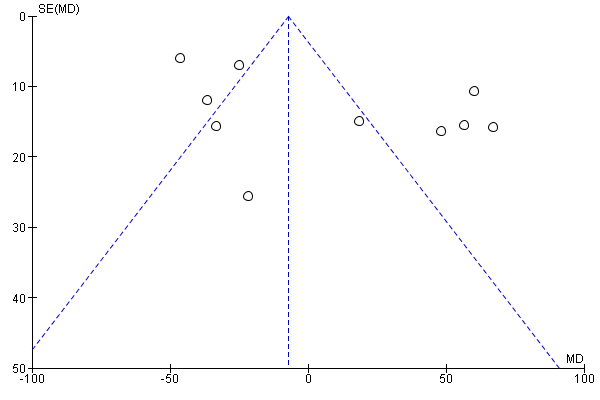


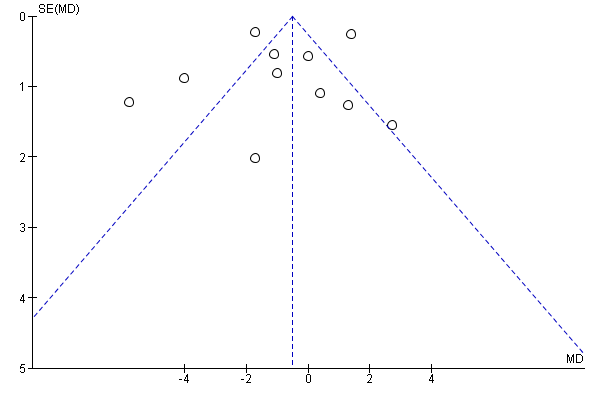


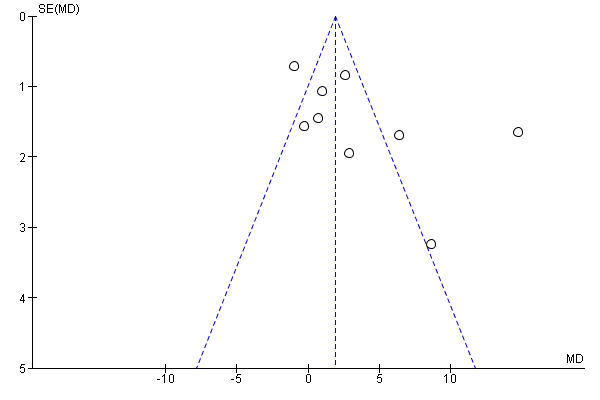


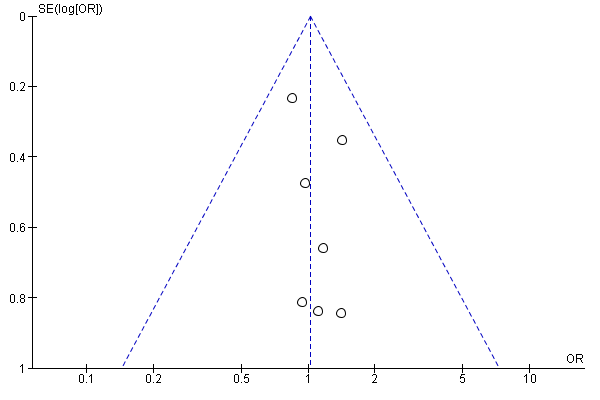


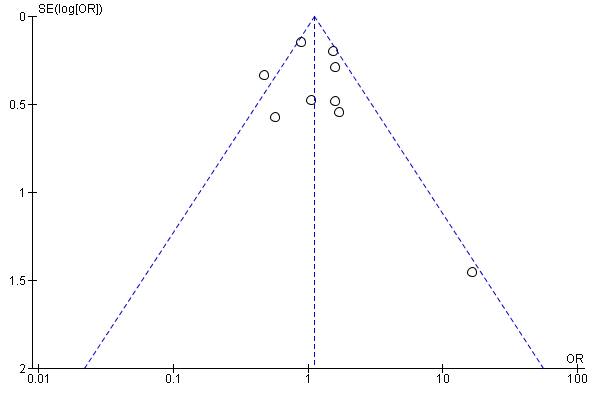

Supplement: Supplementary file 2 — Supplementary file2 (DOCX 183 KB) [file 10147_2021_1972_MOESM2_ESM.docx]
